# Environmental filtering triggers community assembly of forest understorey plants in Central European pine stands

**DOI:** 10.1038/s41598-017-00255-z

**Published:** 2017-03-21

**Authors:** Werner Ulrich, Piotr Sewerniak, Radosław Puchałka, Marcin Piwczyński

**Affiliations:** 1 Chair of Ecology and Biogeography, Nicolaus Copernicus University, Lwowska 1, PL-87-100 Toruń, Poland; 2 Department of Soil Science and Landscape Management, Nicolaus Copernicus University, Lwowska 1, PL-87-100 Toruń, Poland

## Abstract

Habitat filtering models predict ecologically similar plant species to jointly colonize sites due to comparable environmental characteristics leading to an aggregated pattern of species spatial occurrence. Models focused on interspecific competition expect species with similar ecological requirements to be spatially segregated. While both models are corroborated by field work, few empirical studies have tried to infer under which habitat conditions these patterns of co-occurrence prevail. Here we use an exceptional data set on central European pine forest understorey plant communities to assess the change in community structure along gradients of soil productivity and heterogeneity. We found all understorey communities to be significantly nested. The degree of segregation increased with increasing soil Ca and Mg content, as well as with increasing pH, nutrient availability, and moisture. However, variability in soil characteristics did not significantly influence the pattern of species co-occurrence. We also found an intimate link between productivity, species richness, and species segregation making any causal inference challenging. These results point to possible misinterpretations and pitfalls in studies on community assembly. Finally our results demonstrate that managed forests provide a natural experiment of understorey community assembly under controlled conditions, an experiment that deserves further attention.

## Introduction

The question how animal and plant communities assemble has been at the centre of ecological interest since Diamond^1^ proposed his assembly rule model. Theoretical models and empirical work have demonstrated that local (alpha) and regional (gamma) species richness increase with environmental heterogeneity^2, 3^, intransitive (non-hierarchical) competitive interactions^4, 5^, and total species functional trait space^6^. These dependencies are further affected by spatial and temporal variation in community composition (beta diversity)^7, 8^ and the patterns (the geometry) of species co-occurrences^5^ as captured by the meta-community framework^9–11^.

Recent meta-analytical studies have revealed common patterns in this geometry^12–15^. Many meta-communities appear to be nested, that is species poor local communities are true compositional subsets of larger ones^16^. Ulrich *et al*.^14^ identified colonisation - extinction dynamics to be the prevalent cause of a nested community organization. In turn, several contrasting, although not mutually excluding, processes explain non-nested patterns of species occurrences. First, habitat filtering^17^ selects species of similar habitat requirements to jointly colonize sites due to comparable environmental characteristics, leading to an aggregated (modular) pattern of species spatial occurrence. Second, habitat fragmentation^18^, low dispersal^19^, strong competitive interactions^20^, pronounced environmental gradients^21^, and particularly environmental and landscape heterogeneity^22, 23^ counteract the formation of a modular community organization towards a segregated pattern of species co-occurrences with a high degree of spatial species turnover (beta diversity).

Both processes and the respective underlying theories have been tested extensively (reviewed in refs 12 and 17). In plant communities filter effects are particularly important during early succession^23^. The structuring effect of filters are most obvious at spatial scales above the individual interaction horizons (e.g. ref. 17). Competitive interactions, in turn, were found to structure species co-occurrences at small spatial scales^5^ containing sets of subdominant species^24^, in closed communities with stable species composition^25^, and under stable environmental conditions^26^.

However, few works have tried to infer the local variation in the geometry of species occurrences from underlying environmental conditions. Such studies need multiple local inventories and associated environmental data, in the case of plants particularly data on soil mineral and nutrient availability, as well as microclimatic conditions. Most work instead used large-scale biogeographic data to infer changes in community structure along broadly defined environmental, mainly climatic gradients^8, 27^. Importantly, as García-Bacquero and Crujeiras^28^ highlight, a sound assessment of occurrence geometry needs information on multiple environmental clues as these often act in opposite directions.

**Figure 1:**
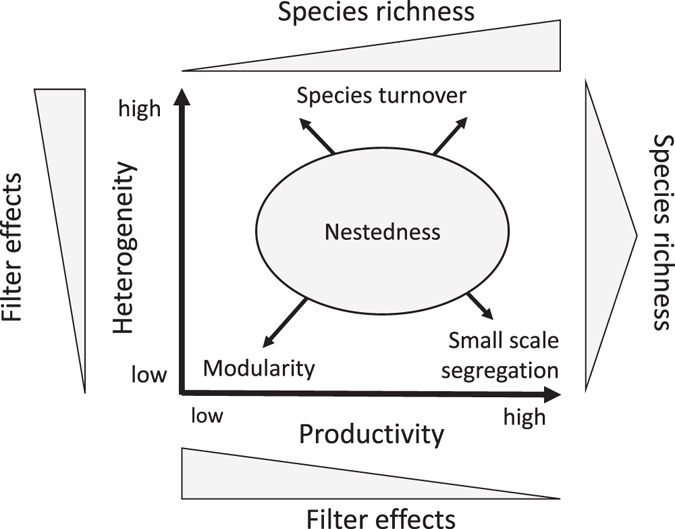
The interplay of environmental heterogeneity and productivity influences the spatial geometry of species occurrences and of species richness. High productivity and low heterogeneity increase the importance of interspecific competition leading to a small-scale segregated pattern of species co-occurrences. Low heterogeneity and productivity cause species to concentrate on islands of fertility leading to an aggregated (modular) pattern of occurrence. A high degree of heterogeneity favours species turnover irrespective of the degree of productivity, while intermediate degrees of both variables should be associated with a nested pattern. Therefore, nestedness is expected to occur particularly in species rich meta-communities at intermediate degrees of habitat filtering.

Based on competition and habitat filtering theory we expected to see occurrence geometry to vary systematically along gradients of soil productivity and environmental variability (Fig. 1). This leads to five basic starting hypotheses. As competitive effects should have the strongest impact on community assembly at low environmental variability^26^ and intermediate productivity (reviewed in ref. 29), we (1) assume that respective communities are most prone to showing competition induced species segregation. In turn, strong environmental filtering is often associated with variable habitat conditions among local patches and (2) should lead to species turnover (e.g. ref. 30). This pattern is equivalent to the well-known distance decay in compositional similarity^31^. In fairly unproductive stable environments suitable habitat patches form strong filters possibly (3) leading to a small scale patchy distribution of species. This is equivalent to an aggregated (modular) pattern of species co-occurrences (Fig. 1).

Nestedness and species segregation (reciprocal exclusions and species turnover) are often seen to be contrasting patterns (e.g. ref. 32). This raises the question under which environmental conditions a nested pattern is expected. Previous work has linked nestedness particularly to high degrees of dispersal and variable community composition (the mass effect model^33^). Given that our model (Fig. 1) expects segregation to be associated with highly variable environmental conditions and with high productivity we (4) hypothesize that a nested meta-community organization should most often be found at intermediate habitat conditions and productivity (Fig. 1). As these are linked to high species richness by the intermediate disturbance hypothesis^34^ we also (5) predict nestedness to occur particularly in species rich meta-communities (cf. ref. 35 for a similar finding in seed disperser networks).

**Figure 2:**
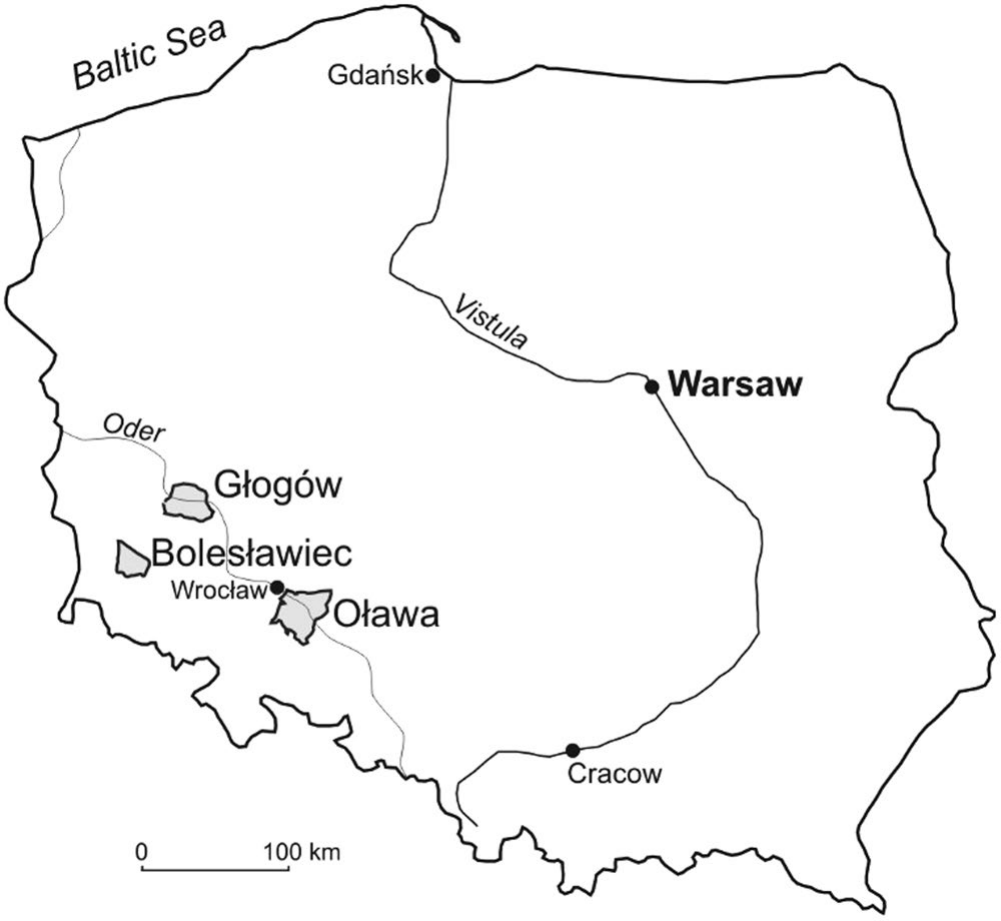
Forests included in the present study (Puchałka unpubl. using DIVA GIS 7.5, http://www.diva-gis.org/).

Below we take the opportunity to test these predictions and the relative influences of habitat characteristics and environmental variability on the composition of local understorey plant communities using an extraordinary data set obtained during recent quantitative surveys of three Polish pine forest plantations (Sewerniak, unpublished and refs 36, 37 and 38). These surveys contain detailed data on soil conditions and complete understorey species lists from 130 forest plots (Fig. 2). Understories contribute to forest nutrient cycles^39^, productivity^40^, and regeneration ability^41^ and consequently have a major, although often neglected, impact on forest management. Indeed, recent work has highlighted the importance of understorey plants on vegetation recovery^42, 43^.

## Results

**Figure 3:**
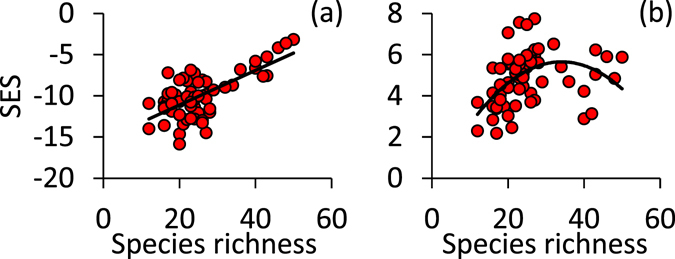
Dependences of standardized effects sizes (SES based on the equiprobable-equiprobable null model) of the C-score (**a**) and NODF (**b**) on species richness. Linear regression in (**a**) Linear OLS regression in a: r^2^ = 0.46 (P(F_1,61_) < 0.001, quadratic OLS regression in b: r^2^ = 0.21 (P(F_1,61_) < 0.01.

Plot species richness increased with increasing moisture and pH and to a smaller degree with decreasing C/N ratio in the soil humus horizon (Table 1). Forest division and age of stands, but also nutrient demands did not significantly influence richness (Table 1). All of the meta-communities were significantly nested (Fig. 3) with respect to an equiprobable random expectation and consequently none of them exhibited a segregated pattern (Fig. 3a). With increasing species richness the degree of species segregation changed towards random (Fig. 3a). In contrast, Akaike model comparison indicated that the degree of nestedness was highest at intermediate species richness (Fig. 3b, r^2^ = 0.21, AICc = 84.3) in comparison with a linear increase (r^2^ = 0.07, AICc = 96.1).

**Table 1:** Generalized linear modelling pointed to pH, moisture, and to a lesser degree C/N ratio as main predictors of plot ln-transformed species richness.

Variable	df	partial η^2^	Beta ± standard error
N	1	0.02	0.20 ± 0.12
C/N	1	0.04	−0.20 ± 0.09
Mg	1	<0.01	−0.14 ± 0.25
Ca	1	<0.01	0.07 ± 0.30
pH	1	0.11**	0.55 ± 0.14
Nutrients	1	<0.01	−0.08 ± 0.13
Moisture	1	0.08*	0.24 ± 0.08
Age	1	0.01	0.05 ± 0.06
Forest stand	2	0.01	
Error	119		

Moisture and pH refer to respective Ellenberg scores. Parametric Bonferroni corrected (nine single tests) significance levels: *P(F) < 0.05, **P(F) < 0.01. Whole model r^2^ = 0.24, P < 0.001.

**Figure 4:**
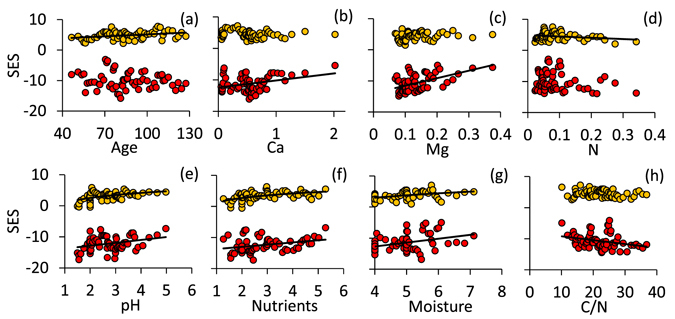
Dependences of standardized effects sizes (SES based on the equiprobable-equiprobable null model) of the C-score (red dots) and NODF (yellow dots) on plot average tree age (**a**), total Ca (**b**), Mg (**c**), and N (**d**) content, Ellenberg value based indices for light (**e**), nutrient (**f**), and moisture demands (**g**), and soil C/N ratios (**h**). Linear OLS regressions of the C-score in (**b**) r^2^ = 0.11 (P(F) < 0.01, (**c**) r^2^ = 0.30 (P(F) < 0.001, (**e**) r^2^ = 0.11 (P(F) < 0.01, (**f**) r^2^ = 0.10 (P(F) < 0.05, (**f**) r^2^ = 0.13 (P(F) < 0.01, (**h**) r^2^ = 0.11 (P(F) < 0.01. Logarithmic OLS regressions of NODF in (**a**) r^2^ = 0.12 (P(F) < 0.01, (**d**) r^2^ = 0.06 (P(F) < 0.10, (**e**) r^2^ = 0.24 (P(F) < 0.001, (**f**) r^2^ = 0.27 (P(F) < 0.001, (**g**) r^2^ = 0.18 (P(F) < 0.01.

The degree of spatial species segregation increased with increasing soil Ca (Fig. 4b) and Mg (Fig. 4c) content, as well as with increasing pH (Fig. 4e), nutrient (Fig. 4f), and moisture (Fig. 4g) demands. Species segregation decreased with increasing C/N ratio (Fig. 4h). In turn the degree of nestedness increased with stand age (Fig. 4a), pH (Fig. 4e) and nutrient (Fig. 4f) and moisture demands (Fig. 4g). The level of nestedness decreased with increasing soil nitrogen content (Fig. 4d). After accounting for the co-variance with species richness and spatial autocorrelation (Table 2), the degree of species spatial segregation was still significantly influenced by soil nitrogen content and moisture demands, and to a lesser degree by pH (Table 2). In turn, the degree of nestedness increased with productivity (Table 2), although this increase was not significant at the 5% error level (Table 2) and explained at most 8% of variance in the standardized effect size of NODF. The degree of nestedness increased with increasing species richness (OLS r^2^ = 0.28). We did not find an indication for the highest NODF at intermediate richness (quadratic OLS r^2^ = 0.30, quadratic term not significant at p < 0.05, (ΔAICc < 1).

**Table 2:** Generalized linear modelling (partial η^2^ values) points to soil nitrogen content, pH and moisture to directly influence the pattern of understorey plant segregation (SES C-score).

Predictor	Age	Ca	Mg	N	pH	Moisture	Nutrients	C/N
Species segregation								
SV	0.01	0.03	0.02	0.23**	0.09	0.20**	0.03	0.02
SV^2^	0.01	0.05	0.04	0.10	0.08	0.19*	0.05	0.01
CV(SV)	<0.01	0.01	0.03	<0.01	0.04	0.03	0.03	0.03
Species	0.42***	0.29***	0.26***	0.51***	0.19*	0.37***	0.11*	0.42***
PCA1	0.06	0.08	0.06	0.04	<0.01	<0.01	0.17*	<0.01
r^2^	0.42***	0.40***	0.45***	0.50***	0.23**	0.41***	0.46***	0.45***
Nestedness								
SV	0.07	<0.01	0.01	0.01	<0.01	0.01	0.04	0.01
SV^2^	0.05	0.03	<0.01	0.02	<0.01	0.01	0.05	<0.01
CV(SV)	0.07	0.08	0.01	<0.01	0.01	0.02	<0.01	<0.01
Species	0.11	0.15	0.24***	0.12*	0.26***	<0.01	0.19*	0.11
PCA1	0.01	0.01	<0.01	0.07*	0.03	0.03	<0.01	0.01
r^2^	0.30**	0.23**	0.31***	0.23**	0.49***	0.18**	0.49***	0.07
Compositional similarity								
SV	0.03	0.03	0.01	0.07	0.03	0.01	0.02	<0.01
SV^2^	0.02	0.01	<0.01	0.03	0.03	0.01	0.03	<0.01
CV(SV)	0.03	0.01	0.01	0.03	<0.01	<0.01	0.04	<0.01
Species	0.40***	0.37***	0.38***	0.50***	0.22**	0.19**	0.54***	0.54***
PCA1	0.02	0.01	<0.01	0.07*	0.01	0.01	0.04	<0.01
r^2^	0.45***	0.49***	0.65***	0.56***	0.65***	0.64***	0.80***	0.64***

Degrees of nestedness (SES NODF) and species composition (SES Soerensen index) were mainly influenced by variation in species richness. Note that SES C-score, SES NODF, and SES Soerensen index served always as dependent variables. SV, squared SV, and the coefficient of variation CV(SV) refer to the respective soil variables listed in the columns. ln-transformed species richness and the dominant spatial eigenvector (PCA1) served as additional covariates. Nutrients, pH, and moisture refer to Ellenberg scores. Parametric Bonferroni corrected (40 single tests) significance levels: *P(F) < 0.05, **P(F) < 0.01, ***P(F) < 0.001.

Compositional similarity as quantified by the Soerensen metric decreased with increasing soil productivity (Supplement Fig. S2a). As in the case of nestedness this pattern was largely due to the covariance with species richness (Table 2). After accounting for richness effects and spatial autocorrelation compositional similarity was not significantly influenced by soil variables except for a weak effect of nitrogen content. Variability in productivity did not significantly influence the geometry of species occurrences (Table 2) except for weak effects of stand age and soil calcium content.

## Discussion

To date most studies on the variability in the pattern of species co-occurrences have focused on changes in beta diversity along biogeographic gradients (reviewed in refs 7 and 44) and on temporal changes in species co-occurrences^45^. At a fine spatial grain, Nguyen and Gómez-Zurita^46^ found weak environmental gradients to be sufficient to trigger high species turnover in tropical beetle communities. Bar-Massada and Belmaker^47^ reported changes in the pattern of tree species co-occurrences from species segregation at optimal to aggregation in poor environmental conditions. Our study is apparently the first that directly relates the spatial patterning of understorey plant species to gradients in environmental conditions within a mature, albeit managed, ecosystem.

All local communities appeared to be less variable in species composition than expected from an equiprobable random assembly (Fig. 4). Additionally, compositional similarity among plots was much higher than expected from the null model (Fig. S2a). This low variability (beta diversity) is a strong sign that local colonizers pass filters leading to communities of similar species composition. Thus our results indicate habitat filtering to be the dominant driver of community assembly while competitive interactions have only a minor impact at least at the present level of spatial resolution, that is within an area of 20 × 20 m^2^. These findings contrast with previous studies on community assembly in comparable plant communities that reported increasing effects of competitively driven species segregation at small spatial scales (e.g. refs 23 and 48) and significant species turnover along environmental gradients at larger scales (ref. 31, but see ref. 47). These diverging results are most parsimoniously explained by the differences in successional stage. Most prior work (e.g. ref. 49) studied early and mid-successional input-driven, open, often grassland, communities where most new colonizers were immediately eliminated by competitively superior resident species. The understorey plant communities of our forest plots, in turn, exhibit pronounced species exchange within this habitat type but not from outside. Thus, resident species have already passed the respective forest habitat filters and competitively inferior species, being less adapted, have already been eliminated. Both processes have probably led to higher compositional similarity than expected from random colonization.

Our first starting hypothesis assumed that low environmental variability and high productivity favour species spatial segregation driven by competition. In line with this expectation, Bar-Massada and Belmaker^47^ found segregated tree occurrences only at favourable environmental conditions. Consequently, we expected to see significant effects of soil C/N ratios, nutrient availability, moisture, and pH as these are directly related to plant growth (reviewed in ref. 50). This was indeed the case. Standardized effect sizes of the C-score increased with increasing nutrient availability, Ca, Mg, moisture, and pH, and decreased with increasing C/N ratio (Fig. 4). Consequently, compositional similarity showed the opposite trend (Fig. S2a). Species segregation is generally expected to increase with increasing competitive interactions for limiting resources^51^. Our results point to small scale variability in habitat conditions as an additional driver of species segregation. A possible mechanism involves respective variability in the major limiting resource resulting in a vicariant pattern of competitive hierarchies. This variability causes a network of competitive strength (competitive intransitivity) as had been found by Soliveres *et al*.^4^ in experimental grassland communities. Importantly, these drivers increase species richness, too (Table 2, ref. 5). It remains a question of interpretation whether the environmental effects on co-occurrence are due to the richness effect or, on the contrary, whether the pattern of co-occurrence triggers increased species richness. Environmental regression analyses alone are not able to answer the underlying cause – effect relationships. We even doubt whether pure field data are able to solve this question. Probably ecologists have to rely on extensive modelling of community assembly to get a testable theoretical background. These models need to include variation in species richness, functional traits, spatial heterogeneity, and competitive interactions. Recently, Ulrich *et al*.^5^ made a first step towards this goal demonstrating that competitive effects in spatially explicit neutral model communities are able to generate different patterns of species co-occurrences depending on species dispersal ability. In a next step these models need to include variability in habitat conditions.

**Figure 5:**
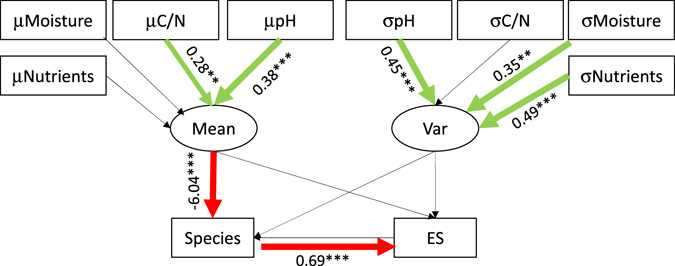
Structural equation modelling including average soil parameters and respective standard deviations (mean (μ) and standard deviation (σ) of nutrient and moisture demands, pH, and C/N ratio) point to a species richness pathway triggering the degree of species spatial segregation (estimated by the effect size *ES* of the equiprobable null model). Parametric statistical support: **P < 0.01; ***P < 0.001. Thickness of arrows is approximately proportional to statistical support of positive (green) and negative (red) influences. Whole model χ^2^ > 100, P (df = 44) < 0.001.

To disentangle the links between environment, richness, and co-occurrence we performed path analytical modelling with the degree of species segregation as target variable (Figs 5 and S2b). Importantly, we did not use the raw value of the C-score that is qualitatively equivalent to the degree of beta diversity leading to the well-known discussion about the relationship between alpha, beta, and gamma diversity^8, 52, 53^. Our target variable was the effect size of segregation after null model comparison and consequently after accounting for the richness effect. Nevertheless, a consistent outcome of this modelling, independent of specific model settings (Fig. S2b), was the richness pathway, that is the strong effect of species richness on segregation. Average soil characteristics triggered richness but not the spatial occurrence geometry (Fig. 5). This result contrasts previous findings (e.g. refs 54 and 55) that reported environmentally induced patterns of species spatial distribution. However, this work analysed the possible direct influence of habitat characteristics on species co-existence without partitioning the effect of species richness. Ulrich *et al*.^56^ tried to disentangle richness and direct environmental effects on plant co-occurrence and were also unable to detect direct soil influences. Clearly, the pathways to specific patterns of spatial distribution need further evaluation.

Surprisingly, the model strongly indicates that environmental variability did not notably affect species richness and the degree of co-occurrence of the present understorey plant communities (Fig. 5, Table 2). Contrary, theory^57^ and empirical data (e.g. refs 58 and 59) point to habitat heterogeneity as being important triggers of plant species segregation mainly due to filter effects for specific environmental niches. However, most work used large scale data on plant occurrences including wide environmental conditions (but see ref. 60). Possibly, environmental variability in our forest plots was below the threshold to induce community wide species segregation.

In this respect we note that the problem of accounting for richness effects is commonly met in species co-occurrence analysis^61^. Null model analysis has been developed to eliminate all effects not directly linked to the pattern of interest^62^. Matrix fill (alpha diversity) and total species richness (gamma diversity) are commonly assumed to be such factors in the study of species co-occurrences (for instance while studying patterns of beta diversity^8, 52^). However, recent theoretical work has shown that richness effects on occurrence geometry cannot be totally removed in statistical analyses^53^. The equiprobable null model used here kept a significant correlation between effect sizes and richness (Fig. 3). On the other side numerical simulations using fractal landscapes^63^ and neutral communities with additional competitive interactions^5^ have pointed to the possibility that species segregation alone might allow for the existence of additional species. Thus increased richness might be the outcome and not the cause of observed patterns of co-occurrences. This latter finding even implies that we might wish to retain occurrence geometry in null model analysis to infer variation in species richness. Indeed, eliminating species richness as a covariate in Table 2 made all metrics of co-occurrences significantly related to at least one of the soil characteristics (not shown) making any causal inference challenging. Consequently, statistical analyses that try to partition richness and turnover effects on community assembly either by variance partitioning^64^ or by null model comparisons^61^ might miss this important inherent link.

Our second and third starting hypotheses assumed environmental filtering within patches in combination with variable habitat conditions among patches and at rather unproductive sites, respectively, to impose species spatial turnover (Fig. 1). At the present level of spatial resolution this was not the case with respect to habitat variability. Observed variability was only marginally (and insignificantly) related to patterns of co-occurrences (Table 2). Apparently the observed range of soil characteristics and the respective variability among patches was not sufficient to induce observable differences in species composition and spatial geometry. In turn, we found corroboration for the productivity hypothesis (Fig. 4). The degree of species segregation significantly increased with microelement and nutrient availability, pH, and moisture, and decreased with increasing C/N ratio. As these variables were also positively linked to species richness (Table 2, Fig. 3) we argue that species spatial segregation and therefore a high degree of spatial turnover are intimately linked to the degree of productivity. Surprisingly, this result contradicts previous findings in grassland communities^65^ where increased seed availability resulted in higher species richness at low but not at high habitat productivity. However, the latter findings were obtained under experimental short-term conditions. As shown here, in the long run highly productive communities should generate increased richness, a finding that is of course in line with the major lines of argument explaining the increase in richness and beta diversity as regards highly productive tropical habitats (reviewed in ref. 7).

We expected to see a nested meta-community organization in intermediate habitat conditions and at intermediate productivity (Fig. 1). This was not the case (Table 2). Nearly all communities were significantly nested and the degree of nestedness increased with increasing productivity. Further, nestedness was highest at intermediate richness (Fig. 3b). Previously, the dependence of the degree of community nestedness on richness and productivity has only been indirectly studied across latitudinal gradients and therefore at larger spatial scales^66^. This work pointed to an increase in species turnover and consequently a decrease in nested community patterns at species rich lower latitudes (e.g. refs 27 and 52).

Our study stands are managed forests with comparably few tree species. Therefore, tree species composition is artificial in comparison to natural forests. However, the understorey plant communities assembled naturally and thus represent the outcome of colonization dynamics and ecological interactions in the situation of comparably homogeneous light and water supplies as realized in mono-stands. In this respect managed forests provide a natural experiment of understorey community assembly under controlled conditions. So far this study system has not received the attention it deserves.

## Methods

### Study sites and sampling

From 2003 to 2005 we studied the Scots pine (*Pinus sylvestris* L.) understorey vegetation of 130 plots (400 m^2^ each) from three Forest Divisions (Bolesławiec, Głogów and Oława) in South-Western Poland (Fig. 2). Soils of the investigated plots covered a wide range both of soil moisture (from dry to boggy) as well as of soil fertility (from poor sandy to fertile fine-textured) and from pine mono-stands to mixed pine forests^36–38^. Respective raw data on the geographic position of each plot, stand age, and variables related to soil fertility are contained in the electronic Supplement Table S1a.

Braun-Blanquet vegetation surveys took place from June to August 2003 to 2005. From these data we compiled a single presence – absence data matrix **M**_total_ containing a total of 101 understorey plant species in rows and 130 plots in columns (Supplementary Material Table S1b).

### Habitat assessment

In each of the 130 studied plots standard humus horizon soil samples were collected. For the present study we estimated soil fertility from the plot average pH, N content, and the respective Carbon- Nitrogen (C/N) ratio, as well as Ca and Mg content^36–38^. Additionally, we used Ellenberg indicator values^67^: pH, moisture, and soil nutrient demands as provided by JUICE 7.0^68^. Average Ellenberg values per site were calculated over all species present at a site (Table S1a). We assessed habitat variability among plots from the coefficients of variation (CV) of these variables.

### Analysis of community assembly

To infer the spatial geometry of species occurrences within the **M**_total_ matrix we used three metrics of species co-occurrences proposed to account for patterns in presence-absence matrices. First, we estimated matrix wide species segregation (negative species associations) implementing the C-score^69^ that is a matrix size normalized count of the number of checkerboard submatrices ({{1, 0}, {0, 1}} or {{0, 1}, {1, 0}}). High C-scores point to species spatial segregation. Second, we applied nestedness analysis^70^ to identify gradients in species co-occurrences and richness across study sites^14^ and to infer which environmental characteristics are linked to these gradients. To assess the degree of nestedness we sorted the presence-absence matrices according to species richness and incidence (marginal totals) and used the commonly applied NODF metric^71^. High NODF values point to a strong nested pattern. We tested for filter effects and shifts in species composition among plots calculating the Soerensen compositional similarity index among all pairs of plots. High values point to compositional similarity.

To relate patterns of co-occurrence among plots to environmental variables we used a sliding window approach and ran a window of six plots (with overlap of two plots) along the **M**_**total**_ matrix. For each window we calculated the Soerensen score, the C-score and NODF as well as the respective environmental variable averages and the coefficient of variation (CV). We ran separate analyses for each environmental variable each time sorting the plots according to this variable from smallest to largest. This procedure ensured that plots of similar variable expression were included in each window (minimizing within window variance with respect to among window variance) allowing for a sufficient discriminant ability.

Raw NODF and C-scores depend on matrix size and fill, and consequently cannot be compared directly^61^. For statistical inference we therefore used a null model approach^61^ and compared the observed metric scores with those obtained from 200 null matrices. As we were mainly interested in the reaction of scores to environmental gradients we applied a randomization that did not constrain occurrence probabilities (the equiprobable -equiprobable null model *EE*). EE accounts for matrix fill and shape but otherwise does not limit the possible metric space avoiding thus the possibility that part of the variance in the co-occurrence metric is taken over by the null model. In the case of the Soerensen index we compared observed similarity per sliding window with that obtained from 200 equiprobable random samples from the regional lower Silesian forest species pool. Each plot sample had the same species richness as observed.

We used standardized effect sizes (*SES* = *Obs* − *Exp)/StDev*_*Exp*_; *Obs* and *Exp*: observed and expected scores, *StDev*_*Exp*_: standard deviation of expectation) in regression analyses. SES scores should have values below −1.96 and above +1.96 at the two-sided 5% error level assuming that the respective null distribution is approximately normal. Based on the above input data we tested our four starting hypotheses using generalized linear fixed effects models (GLM) with identity link function and normal error structure. To account for possible peaks in SES at intermediate productivity we included respective squared variables in the GLM analyses. The pattern of species occurrence entered the model as dependent and the productivity metrics and ln-transformed species richness as independent variables. Due to multiple testing all significant levels were Bonferroni corrected using the number of single tests applied to the same data set as a correction factor. Finally, we explored our hypotheses using structural equation modelling (covariance based SEPATH with maximum likelihood parameter estimation as implemented in Statistica 12.0).

Adjacent plots have a higher probability of containing similar flora than more distant ones. To account for this type of spatial autocorrelation we calculated the dominant eigenvector of the Euclidean distance matrix of the plots based on GPS positioning and used this vector as an additional predictor in the GLMs^72^. This eigenvector should account for the large-scale spatial distribution of the environmental variables. It turned out that the spatial structure of our plots had only a minor influence on the results and explained less than 10% of variances in the co-occurrence. Therefore we could use ordinary least squares (OLS) regression to show the direct dependence of SES scores on species richness and soil variables without the need of additional data transformation. We compared regression models using Akaike information criterion (ΔAICc) and accepted a model as being more informative at |ΔAICc| > 2.

## Electronic supplementary material

Dataset 1

Supplementary Analyses
