# Supplementary material for: Environmental filtering triggers community assembly of forest understorey plants in Central European pine stands
Source: Sci Rep. 2017 Mar 21;7:274. doi: 10.1038/s41598-017-00255-z (PMC5428007; doi:10.1038/s41598-017-00255-z)
Supplement: Supplementary file 2 — Supplementary Analyses [file 41598_2017_255_MOESM2_ESM.doc]

**Supplement S2**

**Environmental filtering triggers community assembly of forest understorey plants in Central European pine stands**

Werner Ulrich, Piotr Sewerniak, Radosław Puchałka, Marcin Piwczyński

**Figure S2a.** Dependences of standardised effects sizes (SES based on the equiprobable-equiprobable null model) of the Soerensen similarity scores on plot average tree age (a), total Ca (b), Mg (c), and N (d) content, Ellenberg value based indices for light (e), nutrient (f), and moisture demands (g), and soil C/N ratios (h). Logarithmic OLS regressions in c: r^2^ = 0.31 (P(F) < 0.001, e : r^2^ = 0.44 (P(F) < 0.001, f : r^2^ = 0.31 (P(F) < 0.001, g: r^2^ = 0.41 (P(F) < 0.001, h: r^2^ = 0.28 (P(F) < 0.001.

**Figure S2b.** Structural equation modelling including average soil parameters and respective standard deviations (mean (μ) and standard deviation (σ) of nutrient and moisture demands, pH, C/N ratio) pointed to a species richness pathway triggering the degree of species spatial segregation (estimated by the standardized effect size S*ES* of the equiprobable null model). Parametric statistical support: ** P < 0.01; *** P < 0.001. Thickness of arrows is approximately proportional to statistical support of positive (green) and negative (red) influences. Whole model χ^2^ > 100, P (df = 44) < 0.001.
